# Crystal structure of (*E*)-*N*′-(3-fluoro-2-hy­droxy­benzyl­idene)isonicotinohydrazide

**DOI:** 10.1107/S2056989017009926

**Published:** 2017-07-11

**Authors:** Suwadee Jiajaroen, Kittipong Chainok, Filip Kielar

**Affiliations:** a Department of Chemistry, Faculty of Science and Technology, Thammasat University, Khlong Luang, Pathum Thani 12121, Thailand; b Materials and Textile Technology, Faculty of Science and Technology, Thammasat University, Khlong Luang, Pathum Thani 12121, Thailand; c Department of Chemistry and Center of Excellence in Biomaterials, Faculty of Science, Naresuan University, Muang, Phitsanulok 65000, Thailand

**Keywords:** crystal structure, iron chelator, isonicotinohydrazide, hydrogen bonding

## Abstract

In the title compound, C_13_H_10_FN_3_O_2_, the mol­ecule has an *E* conformation with respect to the C=N bond of the hydrazone bridge. The dihedral angle between the isonicotinoyl and fluoro­phenol moieties is 4.03 (4)°, and an intra­molecular O—H⋯N hydrogen bond generates an *S*(6) ring motif. In the crystal, mol­ecules are linked by N—H⋯N and C—H⋯N hydrogen bonds, forming chains propagating along the *a*-axis direction. The chains are linked by C—H⋯O hydrogen bonds, resulting in the formation of layers lying parallel to the *ab* plane. The crystal structure also features π–π inter­actions [centroid-to-centroid distance = 3.6887 (8) Å].

## Abstract 2

The title isonicotinohydrazide derivative is planar, with an r.m.s. deviation for the fitted non-H atoms of 0.062 Å, and an intra­molecular O—H⋯N hydrogen bond with an *S*(6) ring motif. In the crystal, mol­ecules are linked by N—H⋯N and C—H⋯N hydrogen bonds forming chains propagating along the *a*-axis direction.

## Chemical context   

Hydrazone-based chelators of metal ions are inter­esting compounds that receive a significant amount of inter­est (Bendová *et al.*, 2010 ▸; Jansová *et al.*, 2014 ▸; Hrušková *et al.*, 2016 ▸). We have recently published the structures of two derivatives of the prototypical chelator from this class, salicyl aldehyde isonicotinoyl hydrazide (SIH), which were synthesized as potential sensors for metal ions (Chainok *et al.*, 2016 ▸). The structures reported have fluorine and methyl substitution in position 5 on the benzene ring. Herein, we report the crystal structure of a further analogue in this series bearing a fluorine substituent in position 3 of the benzene ring, which was synthesized in order to investigate the effect of the distance between the reporting fluorine atom and the metal chelating unit.
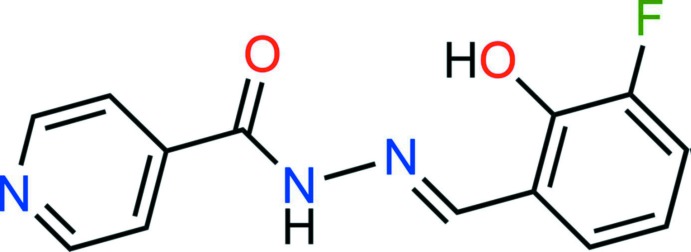


## Structural commentary   

**Figure 1:**
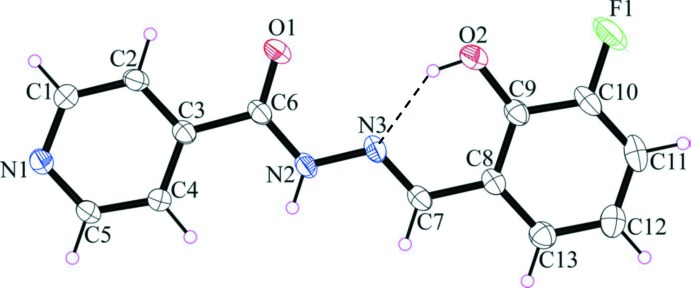
The mol­ecular structure of the title compound, with the atom labelling and 50% probability displacement ellipsoids. The intra­molecular hydrogen bond is shown as a dashed line (see Table 1 ▸).

The mol­ecular structure of the title compound, with atom labelling, is presented in Fig. 1 ▸. The mol­ecule has an *E* conformation with respect to the hydrazone bridge (C7=N3). The C6—N2 and C7—N3 bond lengths differ by 0.08 Å hence; these two bonds are formally double and single bonds, respectively. The mol­ecule deviates slightly from planarity with an r.m.s deviation for the fitted non-hydrogen atoms of 0.062 Å. There is an intra­molecular O2—H2*O*⋯N3 hydrogen bond with an *S*(6) ring motif present in the pyridine carboxamide moiety, and the pyridine ring (N1/C1–C5) is approximately coplanar with the amide group (C6(=O1)N2) [dihedral angle = 8.25 (6)°]. The isonicotinoyl moiety (N1/C1–C6/O1/N2) is inclined to the fluoro­phenol moiety (C8-C13/O2/F1) by 4.03 (4)°.

## Supra­molecular features   

**Figure 2:**
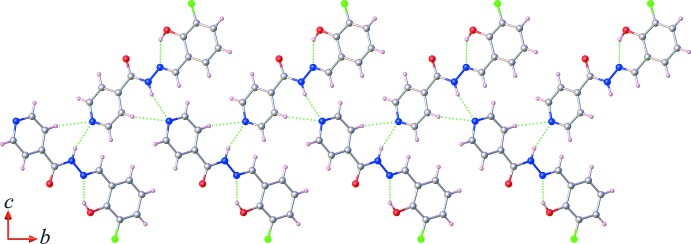
A view of the hydrogen-bonded chains, formed in the crystal structure of the title compound *via* bifurcated-acceptor N—H⋯N and C—H⋯N hydrogen bonds (dashed lines; Table 1 ▸).

**Figure 3:**
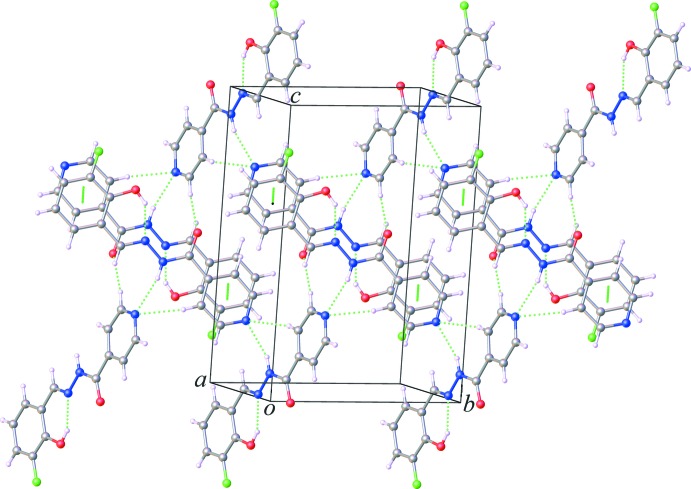
Part of the crystal structure of the title compound, showing the formation of the layers, parallel to the *ab* plane, formed *via* C—H⋯O hydrogen bonds, and the π–π inter­actions (dashed lines).

In the crystal, mol­ecules are linked by bifurcated-acceptor N2—H2*N*⋯N1^i^ and C4—H4⋯N1^i^ hydrogen bonds (Table 1 ▸), leading to the formation of zigzag chains lying parallel to the *b*-axis direction, as shown in Fig. 2 ▸. Adjacent chains are further linked *via* C5—H5⋯O1^ii^ hydrogen bonds, forming layers parallel to the *ab* plane, as shown in Fig. 3 ▸. Within the sheets, there are π–π stacking inter­actions involving inversion-related mol­ecules [*Cg*1⋯*Cg*2^i^ = 3.6887 (8) Å; *Cg*1 and *Cg*2 are the centroids of the pyridine (N1/C1–C5) and phenyl (C8–C13) rings, respectively; symmetry code: (i) −*x* + 1, −*y* + 1, −*z* + 1].

## Database survey   

A search of the Cambridge Structural Database (Version 5.38, latest update May 2017; Groom *et al.*, 2016 ▸) for compounds with the (*E*)-*N*-(2-hy­droxy­benzyl­idene)isonicotinohydrazide skeleton revealed 86 hits. They include the isotypic crystal structures with bromide (PORYEC; Xiong & Li, 2014 ▸), meth­oxy (CANCOK, Yu *et al.*, 2005 ▸; CANCOK01, Yang, 2007 ▸; CANCOK02, Xu, 2013 ▸), and hy­droxy (WAFVEG; Tecer *et al.*, 2010 ▸) groups substituted at the 3-position of the phenyl ring.

## Synthesis and crystallization   

Isonicotinic acid hydrazide (301 mg, 2.19 mmol) and 3-fluoro­salicyl­aldehyde (338 mg, 2.69 mmol) were suspended in a 1:1 mixture of water and ethanol (6 ml). The reaction mixture was stirred at 363 K for 24 h and formation of a precipitate was observed. The reaction mixture was allowed to cool to room temperature and then filtered. The isolated solid was washed with water to give the product as a white solid (510 mg, 1.97 mmol, 90%). Colourless rod-like crystals, suitable for X-ray diffraction analysis, were grown by slow evaporation of a solution in methanol of the title compound. ^1^H NMR (400 MHz, DMSO-*d*_6_) *d* 6.94 (1H, *m*, CH-Ph), 7.32 (1H, *dd*, *J* = 8.8, *J* = 10.4 CH-Ph), 7.44 (1H, *d*, *J* = 8.4, CH-Ph), 7.85 (2H, *d*, *J* = 5.6, CH-Py), 8.70 (1H, *s*, CH=N), 8.81 (2H, *d*, *J* = 5.6, CH-Py), 11.40 (1H, *s*, NH), 12.39 (1H, *s*, OH). HR–MS (ES^+^) C_13_H_11_FN_3_O_2_ requires 260.0835 [*M* + H]^+^; found 260.0830.

## Refinement   

Crystal data, data collection and structure refinement details are summarized in Table 2 ▸. H atoms bonded to C, N, and O atoms were placed at calculated positions and refined using a riding-model approximation: N—H = 0.86 Å, O—H = 0.82 Å and C—H = 0.93 Å with *U*_iso_(H) = 1.5*U*_eq_(O) and 1.2*U*_eq_(N,C) for other H atoms.

## Supplementary Material

Crystal structure: contains datablock(s) I. DOI: 10.1107/S2056989017009926/su5379sup1.cif

Structure factors: contains datablock(s) I. DOI: 10.1107/S2056989017009926/su5379Isup2.hkl

Supporting information file. DOI: 10.1107/S2056989017009926/su5379Isup3.cdx

Supporting information file. DOI: 10.1107/S2056989017009926/su5379Isup4.cml

CCDC reference: 1560196

Additional supporting information:  crystallographic information; 3D view; checkCIF report

## Figures and Tables

**Table 1:** Hydrogen-bond geometry (Å, °)

*D*—H⋯*A*	*D*—H	H⋯*A*	*D*⋯*A*	*D*—H⋯*A*
O2—H2*O*⋯N3	0.82	1.87	2.5862 (14)	145
N2—H2*N*⋯N1^i^	0.86	2.16	2.9851 (16)	161
C4—H4⋯N1^i^	0.93	2.51	3.3492 (18)	151
C5—H5⋯O1^ii^	0.93	2.37	3.2738 (17)	163

Symmetry codes: (i) 
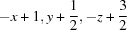
; (ii) 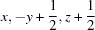.

**Table 2:** Experimental details

Crystal data	
Chemical formula	C_13_H_10_FN_3_O_2_
*M*_r_	259.24
Crystal system, space group	Monoclinic, *P*2_1_/*c*
Temperature (K)	296
*a*, *b*, *c* (Å)	7.8555 (3), 10.2748 (5), 14.9390 (7)
β (°)	92.397 (2)
*V* (Å^3^)	1204.73 (9)
*Z*	4
Radiation type	Mo *K*α
μ (mm^−1^)	0.11
Crystal size (mm)	0.28 × 0.14 × 0.14

Data collection	
Diffractometer	Bruker D8 QUEST CMOS
Absorption correction	Multi-scan (*SADABS*; Bruker, 2016 ▸)
*T*_min_, *T*_max_	0.700, 0.745
No. of measured, independent and observed [*I* > 2σ(*I*)] reflections	22872, 2475, 1769
*R*_int_	0.045
(sin θ/λ)_max_ (Å^−1^)	0.625

Refinement	
*R*[*F* ^2^ > 2σ(*F* ^2^)], *wR*(*F* ^2^), *S*	0.038, 0.100, 1.04
No. of reflections	2475
No. of parameters	173
H-atom treatment	H-atom parameters constrained
Δρ_max_, Δρ_min_ (e Å^−3^)	0.16, −0.17

Computer programs: *APEX3* and *SAINT* (Bruker, 2016 ▸), *SHELXT* (Sheldrick, 2015*a* ▸), *SHELXL2016* (Sheldrick, 2015*b* ▸) and *OLEX2* (Dolomanov *et al.*, 2009 ▸).

## supplementary crystallographic information

### Crystal data

**Table:**

C_13_H_10_FN_3_O_2_	*F*(000) = 536
*M**_r_* = 259.24	*D*_x_ = 1.429 Mg m^−^^3^
Monoclinic, *P*2_1_/*c*	Mo *K*α radiation, λ = 0.71073 Å
*a* = 7.8555 (3) Å	Cell parameters from 5896 reflections
*b* = 10.2748 (5) Å	θ = 3.3–26.2°
*c* = 14.9390 (7) Å	µ = 0.11 mm^−^^1^
β = 92.397 (2)°	*T* = 296 K
*V* = 1204.73 (9) Å^3^	Rod, light colourless
*Z* = 4	0.28 × 0.14 × 0.14 mm

### Data collection

**Table:**

Bruker D8 QUEST CMOS diffractometer	2475 independent reflections
Radiation source: microfocus sealed x-ray tube, Incoatec Iµus	1769 reflections with *I* > 2σ(*I*)
Graphite Double Bounce Multilayer Mirror monochromator	*R*_int_ = 0.045
Detector resolution: 10.5 pixels mm^-1^	θ_max_ = 26.4°, θ_min_ = 3.3°
φ and ω scans	*h* = −9→9
Absorption correction: multi-scan (SADABS; Bruker, 2016)	*k* = −12→12
*T*_min_ = 0.700, *T*_max_ = 0.745	*l* = −18→18
22872 measured reflections	

### Refinement

**Table:**

Refinement on *F*^2^	Primary atom site location: structure-invariant direct methods
Least-squares matrix: full	Secondary atom site location: difference Fourier map
*R*[*F*^2^ > 2σ(*F*^2^)] = 0.038	Hydrogen site location: inferred from neighbouring sites
*wR*(*F*^2^) = 0.100	H-atom parameters constrained
*S* = 1.04	*w* = 1/[σ^2^(*F*_o_^2^) + (0.0468*P*)^2^ + 0.1698*P*] where *P* = (*F*_o_^2^ + 2*F*_c_^2^)/3
2475 reflections	(Δ/σ)_max_ < 0.001
173 parameters	Δρ_max_ = 0.16 e Å^−^^3^
0 restraints	Δρ_min_ = −0.17 e Å^−^^3^

### Special details

**Table:**

Geometry. All esds (except the esd in the dihedral angle between two l.s. planes) are estimated using the full covariance matrix. The cell esds are taken into account individually in the estimation of esds in distances, angles and torsion angles; correlations between esds in cell parameters are only used when they are defined by crystal symmetry. An approximate (isotropic) treatment of cell esds is used for estimating esds involving l.s. planes.

### Fractional atomic coordinates and isotropic or equivalent isotropic displacement parameters (Å^2^)

**Table:**

	*x*	*y*	*z*	*U*_iso_*/*U*_eq_	
F1	0.04982 (16)	0.68426 (11)	0.21940 (6)	0.0860 (4)	
O1	0.42340 (15)	0.25717 (10)	0.46852 (6)	0.0554 (3)	
O2	0.19539 (16)	0.52442 (11)	0.33911 (7)	0.0600 (3)	
H2O	0.2346	0.4824	0.3818	0.090*	
N1	0.63170 (16)	0.03844 (11)	0.75111 (8)	0.0441 (3)	
N2	0.34254 (15)	0.40224 (11)	0.57234 (7)	0.0403 (3)	
H2N	0.3409	0.4242	0.6279	0.048*	
N3	0.27004 (15)	0.47948 (11)	0.50651 (8)	0.0399 (3)	
C1	0.6525 (2)	0.01892 (14)	0.66412 (10)	0.0468 (4)	
H1	0.7160	−0.0527	0.6473	0.056*	
C2	0.58528 (18)	0.09868 (13)	0.59763 (9)	0.0412 (3)	
H2B	0.6029	0.0803	0.5378	0.049*	
C3	0.49156 (17)	0.20612 (12)	0.62066 (8)	0.0351 (3)	
C4	0.4691 (2)	0.22719 (14)	0.71071 (9)	0.0446 (4)	
H4	0.4061	0.2981	0.7293	0.054*	
C5	0.5408 (2)	0.14213 (14)	0.77255 (9)	0.0463 (4)	
H5	0.5248	0.1581	0.8329	0.056*	
C6	0.41696 (18)	0.28991 (13)	0.54677 (9)	0.0386 (3)	
C7	0.18964 (18)	0.58148 (13)	0.52892 (9)	0.0388 (3)	
H7	0.1809	0.6025	0.5891	0.047*	
C8	0.11173 (17)	0.66478 (13)	0.46000 (9)	0.0369 (3)	
C9	0.11849 (19)	0.63208 (13)	0.36907 (9)	0.0415 (3)	
C10	0.0421 (2)	0.71659 (16)	0.30725 (10)	0.0524 (4)	
C11	−0.0379 (2)	0.82835 (16)	0.33089 (11)	0.0575 (5)	
H11	−0.0862	0.8831	0.2872	0.069*	
C12	−0.0460 (2)	0.85917 (15)	0.42025 (11)	0.0541 (4)	
H12	−0.1014	0.9346	0.4374	0.065*	
C13	0.02768 (19)	0.77830 (14)	0.48399 (10)	0.0468 (4)	
H13	0.0214	0.7996	0.5443	0.056*	

### Atomic displacement parameters (Å^2^)

**Table:**

	*U*^11^	*U*^22^	*U*^33^	*U*^12^	*U*^13^	*U*^23^
F1	0.1259 (10)	0.0961 (8)	0.0341 (5)	0.0010 (7)	−0.0206 (5)	0.0070 (5)
O1	0.0834 (8)	0.0553 (7)	0.0275 (5)	0.0052 (6)	0.0002 (5)	0.0019 (5)
O2	0.0887 (9)	0.0545 (7)	0.0356 (6)	0.0095 (6)	−0.0102 (6)	−0.0071 (5)
N1	0.0577 (8)	0.0394 (7)	0.0347 (6)	0.0017 (6)	−0.0025 (5)	0.0029 (5)
N2	0.0548 (7)	0.0375 (6)	0.0279 (6)	−0.0019 (5)	−0.0078 (5)	0.0044 (5)
N3	0.0493 (7)	0.0360 (6)	0.0336 (6)	−0.0058 (5)	−0.0088 (5)	0.0069 (5)
C1	0.0592 (10)	0.0391 (8)	0.0422 (8)	0.0079 (7)	0.0015 (7)	−0.0014 (6)
C2	0.0530 (9)	0.0413 (8)	0.0293 (7)	−0.0019 (7)	0.0027 (6)	−0.0033 (6)
C3	0.0411 (7)	0.0334 (7)	0.0304 (7)	−0.0078 (6)	−0.0014 (5)	0.0006 (5)
C4	0.0631 (10)	0.0387 (8)	0.0320 (7)	0.0094 (7)	0.0016 (6)	−0.0010 (6)
C5	0.0682 (10)	0.0433 (8)	0.0273 (7)	0.0049 (7)	0.0003 (7)	−0.0001 (6)
C6	0.0477 (8)	0.0391 (8)	0.0287 (7)	−0.0074 (6)	−0.0012 (6)	0.0042 (6)
C7	0.0472 (8)	0.0397 (7)	0.0291 (7)	−0.0090 (7)	−0.0049 (6)	0.0027 (6)
C8	0.0391 (7)	0.0361 (7)	0.0348 (7)	−0.0090 (6)	−0.0046 (6)	0.0042 (6)
C9	0.0498 (9)	0.0391 (7)	0.0347 (7)	−0.0084 (7)	−0.0069 (6)	0.0019 (6)
C10	0.0639 (10)	0.0603 (10)	0.0318 (7)	−0.0106 (8)	−0.0127 (7)	0.0084 (7)
C11	0.0568 (10)	0.0530 (10)	0.0610 (11)	−0.0064 (8)	−0.0178 (8)	0.0228 (8)
C12	0.0529 (9)	0.0446 (9)	0.0641 (11)	0.0022 (7)	−0.0057 (8)	0.0082 (8)
C13	0.0511 (9)	0.0451 (8)	0.0440 (8)	−0.0039 (7)	−0.0012 (7)	0.0001 (7)

### Geometric parameters (Å, º)

**Table:**

F1—C10	1.3576 (18)	C3—C6	1.4997 (18)
O1—C6	1.2195 (16)	C4—H4	0.9300
O2—H2O	0.8200	C4—C5	1.3753 (19)
O2—C9	1.3459 (18)	C5—H5	0.9300
N1—C1	1.3317 (19)	C7—H7	0.9300
N1—C5	1.3290 (18)	C7—C8	1.4541 (19)
N2—H2N	0.8600	C8—C9	1.4026 (19)
N2—N3	1.3692 (15)	C8—C13	1.394 (2)
N2—C6	1.3559 (18)	C9—C10	1.386 (2)
N3—C7	1.2756 (18)	C10—C11	1.362 (2)
C1—H1	0.9300	C11—H11	0.9300
C1—C2	1.376 (2)	C11—C12	1.376 (2)
C2—H2B	0.9300	C12—H12	0.9300
C2—C3	1.3784 (19)	C12—C13	1.373 (2)
C3—C4	1.3813 (18)	C13—H13	0.9300
			
C9—O2—H2O	109.5	N2—C6—C3	116.17 (11)
C5—N1—C1	116.42 (12)	N3—C7—H7	120.1
N3—N2—H2N	121.2	N3—C7—C8	119.74 (12)
C6—N2—H2N	121.2	C8—C7—H7	120.1
C6—N2—N3	117.53 (11)	C9—C8—C7	120.86 (13)
C7—N3—N2	118.90 (12)	C13—C8—C7	120.01 (12)
N1—C1—H1	118.1	C13—C8—C9	119.13 (13)
N1—C1—C2	123.77 (13)	O2—C9—C8	123.69 (12)
C2—C1—H1	118.1	O2—C9—C10	118.77 (13)
C1—C2—H2B	120.3	C10—C9—C8	117.54 (14)
C1—C2—C3	119.32 (12)	F1—C10—C9	117.07 (15)
C3—C2—H2B	120.3	F1—C10—C11	119.78 (14)
C2—C3—C4	117.38 (12)	C11—C10—C9	123.15 (14)
C2—C3—C6	118.19 (12)	C10—C11—H11	120.5
C4—C3—C6	124.40 (13)	C10—C11—C12	119.08 (14)
C3—C4—H4	120.4	C12—C11—H11	120.5
C5—C4—C3	119.28 (13)	C11—C12—H12	120.1
C5—C4—H4	120.4	C13—C12—C11	119.86 (15)
N1—C5—C4	123.83 (13)	C13—C12—H12	120.1
N1—C5—H5	118.1	C8—C13—H13	119.4
C4—C5—H5	118.1	C12—C13—C8	121.22 (14)
O1—C6—N2	122.72 (12)	C12—C13—H13	119.4
O1—C6—C3	121.11 (13)		
			
F1—C10—C11—C12	179.40 (15)	C4—C3—C6—O1	−170.67 (14)
O2—C9—C10—F1	0.2 (2)	C4—C3—C6—N2	9.1 (2)
O2—C9—C10—C11	−179.55 (14)	C5—N1—C1—C2	−0.3 (2)
N1—C1—C2—C3	0.5 (2)	C6—N2—N3—C7	175.56 (12)
N2—N3—C7—C8	−179.99 (11)	C6—C3—C4—C5	178.64 (13)
N3—N2—C6—O1	1.1 (2)	C7—C8—C9—O2	−0.1 (2)
N3—N2—C6—C3	−178.64 (11)	C7—C8—C9—C10	−179.69 (13)
N3—C7—C8—C9	2.3 (2)	C7—C8—C13—C12	179.56 (13)
N3—C7—C8—C13	−178.24 (12)	C8—C9—C10—F1	179.79 (13)
C1—N1—C5—C4	0.2 (2)	C8—C9—C10—C11	0.1 (2)
C1—C2—C3—C4	−0.5 (2)	C9—C8—C13—C12	−1.0 (2)
C1—C2—C3—C6	−178.83 (13)	C9—C10—C11—C12	−0.9 (3)
C2—C3—C4—C5	0.4 (2)	C10—C11—C12—C13	0.8 (2)
C2—C3—C6—O1	7.5 (2)	C11—C12—C13—C8	0.1 (2)
C2—C3—C6—N2	−172.75 (12)	C13—C8—C9—O2	−179.55 (13)
C3—C4—C5—N1	−0.3 (2)	C13—C8—C9—C10	0.8 (2)

### Hydrogen-bond geometry (Å, º)

**Table:**

*D*—H···*A*	*D*—H	H···*A*	*D*···*A*	*D*—H···*A*
O2—H2*O*···N3	0.82	1.87	2.5862 (14)	145
N2—H2*N*···N1^i^	0.86	2.16	2.9851 (16)	161
C4—H4···N1^i^	0.93	2.51	3.3492 (18)	151
C5—H5···O1^ii^	0.93	2.37	3.2738 (17)	163

Symmetry codes: (i) −*x*+1, *y*+1/2, −*z*+3/2; (ii) *x*, −*y*+1/2, *z*+1/2.
